# Physical activity perceptions, context, barriers, and facilitators from a Hispanic child's perspective

**DOI:** 10.3402/qhw.v11.31949

**Published:** 2016-08-16

**Authors:** Sharon E. Taverno Ross, Lori A. Francis

**Affiliations:** 1 Health and Physical Activity, University of Pittsburgh, Pittsburgh, PA, USA; 2 Department of Biobehavioral Health, Pennsylvania State University, University Park, PA, USA

**Keywords:** Child, exercise, sedentary behavior, immigrant, qualitative research

## Abstract

**Background:**

In order to develop effective physical activity interventions and to address the burden of obesity in Hispanic children, qualitative studies are needed to build descriptive theory and expand the state of the science. The purpose of this study is to describe physical activity perceptions, context, facilitators, and barriers from the perspective of Hispanic immigrant-origin children.

**Method:**

This in-depth, ethnographic study included 14, 6- to 11-year old, first- and second- generation Hispanic children recruited from an afterschool program in Southeastern Pennsylvania, USA. Methods included child observation, field notes, semi-structured interviews, and a PhotoVoice activity. Transcripts and field notes were coded and analyzed using the constant comparison method to identify overarching themes and patterns in the data.

**Results:**

Data analysis yielded four overarching themes regarding children's perspectives on physical activity. Children engaged in a variety of physical activities and sedentary behaviors, which differed by physical (e.g., park, outside home, and afterschool programs) and social (e.g., parents, siblings, and friends) contexts. Children discussed specific benefits of physical activity. Children's negative attitudes toward physical activity were related to physical discomfort, low athletic competence, and safety concerns. Children perceived physical activity and play to be one in the same, and “fun” was identified as a primary driver of physical activity preferences. The facilitators and barriers to physical activity were related to specific parent/home, school, and neighborhood factors.

**Conclusion:**

Findings from this study suggest that an emphasis on fun and active play, while taking into account family and neighborhood context, may be a desirable intervention approach in Hispanic immigrant-origin children. This study lays the groundwork for future studies to further explore some of the themes identified here to better understand children's conceptualization of and experience with physical activity. Such research may inform the design of programs to increase physical activity or active play, and ultimately promote health and well-being, in this at-risk population.

Immigrant-origin children (i.e., foreign-born children and US-born children with at least one foreign-born parent) are the fastest growing segment of the US child population (Suarez-Orozco, Yoshikawa, & Tseng, 2015). Immigrant-origin children may be at risk for poorer health outcomes as they generally live in low-income families and have parents with low education levels and limited English proficiency (Capps, Fix, Ost, Reardon-Anderson, & Passel, 2004; Hernandez, 1999; Suarez-Orozco et al., 2015). Previous studies have demonstrated that first- and second-generation Hispanic children are at an increased risk of obesity and engage in low levels of physical activity (Liu, Probst, Harun, Bennett, & Torres, 2009; Singh, Kogan, & Yu, 2009; Singh, Yu, Siahpush, & Kogan, 2008; Taverno, Rollins, & Francis, 2010). Engaging in regular physical activity has been identified as one way to prevent obesity and other comorbidities (Physical Activity Guidelines Advisory Committee, 2008). As such, there is a pressing need to study the physical activity of Hispanic immigrant-origin children.

Over the last decade, previous studies have started to provide some insight into the behaviors and context surrounding physical activity for Hispanic children in general. For example, one study found that Hispanic child physical activity levels were associated with the mother's physical activity levels (Olvera et al., 2011), and common physical activities included unstructured play (e.g., climbing and outdoor play) and walking (Butte, Puyau, Adolph, Vohra, & Zakeri, 2007). One recent longitudinal study found that some child (e.g., age, gender, and BMI) and parent (e.g., maternal BMI and education, paternal age, and acculturation) factors were determinants of physical activity and sedentary behavior in 8–10-year-old Hispanic children (Butte et al., 2014). However, previous studies have been limited in that few have provided an in-depth look at physical activity in Hispanic immigrant-origin children, and most have relied on parent-proxy report.

Several qualitative studies have explored Hispanic children's perceptions of physical activity primarily using focus groups or individual interviews (Mier et al., 2013; Rawlins, Baker, Maynard, & Harding, 2013; Skelton, Irby, Guzman, & Beech, 2012; Snethen, Hewitt, & Petering, 2007; Taylor et al., 1999; Umstattd Meyer, Sharkey, Patterson, & Dean, 2013). Another study engaged Hispanic teen girls in a community physical activity assessment using PhotoVoice and focus group methodology and found that parental restrictions and work, transportation, and safety issues were barriers to afterschool physical activity (Hannay, Dudley, Milan, & Leibovitz, 2013). However, these approaches lacked the depth and nuances of multiple individual interviews and extended field observation, and did not involve children younger than 8 years of age.

A review of the literature of physical activity interventions for Hispanic youth found that specific behavioral, parental, cultural, and school/community factors may influence their physical activity (Merianos, 2013). However, given the lack of data on first- and second- generation Hispanic children specifically, comprehensive qualitative studies are needed to build descriptive theory and expand the state of the science. The purpose of this study is to describe physical activity perceptions, context, facilitators, and barriers from the perspective of Hispanic immigrant-origin children.

## Research approach and theoretical framework

For the present study, ethnography, a scientific and inductive approach to learning about the social and cultural life of communities, institutions, and other settings, was selected as the research approach (LeCompte & Schensul, 1999). Hallmarks of this approach include describing a culture-sharing group using the researcher as the primary data collection tool (through observation and interviews) and the concept of culture as a lens through which to interpret results (Creswell, 2007). The tools of ethnography are designed for discovery using a data driven approach (i.e., allow the findings to guide the hypotheses and conclusions); in this study, ethnography was used to highlight the Hispanic children of immigrants’ behavior and the ways in which they construct and make meaning of their worlds.

Because physical activity is influenced by multiple factors including demographic, psychological, environmental, and socio-cultural (World Health Organization, 2010), this study also adopted a socioecological framework (Bronfenbrenner, 1979, 1986; Bronfenbrenner & Morris, 1998). The Social Ecological model posits that individuals function within multiple, nested levels, or social contexts that influence their health behaviors (i.e., individual, intrapersonal, and community/societal) (Davison & Birch, 2001; Stokols, 1996). In this in-depth, ethnographic study (LeCompte & Schensul, 1999), the approach and measures were designed to understand Hispanic immigrant-origin children's perceptions of physical activity, including identifying influences on the child-, parent-, and neighborhood level.

## Methods

### Participants and setting

Participants were recruited from an afterschool program targeting low-income, at-risk children in a semi-urban city in Southeastern Pennsylvania. Sample size was informed by a purposeful sampling strategy and ongoing qualitative data analysis; recruitment ended when saturation of themes was reached (Schensul, Schensul, & LeCompte, 1999). Families were recruited in person by the lead researcher and through flyers posted at the afterschool program and interested parents were prompted to contact the study staff. Participants were eligible to participate if: (1) the parent self-identified as Hispanic/Latino, (2) the child was between the ages of 6 and 11 years, (3) at least one parent was born outside of the USA (including Puerto Rico), and (4) the child spoke either English or Spanish. For this study, participants included 14, 6- to 11-year-old Hispanic children. Children were on average 8.4 (±1.7) years old, and the majority (*n*=11) were boys. Eleven children were second generation with at least one foreign-born parent; the remaining first generation children had lived in the USA for less than 4 years. Participant demographic characteristics are summarized in Table I.

**Table I:** Demographic characteristics (m [SD] or %) of *n*=14 Hispanic children.

Characteristic	Total (*n*=14)	First generation^a^ (*n*=3)	Second generation^a^ (*n*=11)
Gender, % male	85.7%	66.7%	91.0%
Age (years)	8.4 (1.7)	9.0 (1.0)	8.2 (1.8)
Immigrant generation			
First	21.4%		
Second	78.6%		
Country of origin			
PR	57.1%	66.7%	54.6%
Dom	21.4%	0.0%	27.3%
Cuba	7.1%	33.3%	0.0%
PR/Dom	14.3%	0.0%	18.2%

a First generation defined as a foreign-born child with at least one foreign-born parent; second generation defined as US-born child with at least one foreign-born parent.

### Ethical considerations

The Institutional Review Board at The Pennsylvania State University reviewed and approved all study procedures. Informed written consent (parents) and verbal assent (child) were obtained from all participants included in the study. Important ethical considerations when conducting research with children (power relations, informed consent, and confidentiality) were also taken into account (Kirk, 2006). Specifically, several measures were put into place in order to protect this vulnerable group: (1) the study information was presented using age-appropriate language, (2) the children were re-assented prior to every study activity and the researcher continually checked that the child desired to continue participating during each activity, (3) interview formats were flexible and allowed for deviation from the original questions and prompts as directed by the children, and (4) we included child-friendly data collection methods (e.g., photo activity).

### Data collection

All study materials (e.g., consent forms, recruitment flyers) were available in both English and Spanish, and all translations and back-translations were performed by two bilingual/bicultural professionals. All children spoke English and understood Spanish, and approximately half spoke Spanish fluently. The researcher was bilingual in English and Spanish, and her native language was English. All children preferred to have the interviews performed in English. Interviews were transcribed verbatim, and the lead researcher verified the interview transcripts against the original audio files. All study materials were de-identified and stored on a password protected computer.

Trustworthiness, rather than generalizability of the study findings, is an important feature of qualitative research (Lincoln & Guba, 1985; Merriam, 1998). This study sought to increase trustworthiness through credibility and transferability. Credibility of the research can be enhanced through (1) rigorous techniques and methods of data collection/analysis and (2) credibility of the researcher (Patton, 1990). This study was completed as part of the dissertation research of the lead researcher. Prior to gaining access to the participants and setting, the researcher had several meetings with key gatekeepers (chief-executive officer and program directors of the afterschool program). Over a period of 4 months, the researcher volunteered daily at the afterschool program where she became a trusted and visible part of the team. To build trust and rapport with the staff and children, the researcher assisted with child transport, daily programming, academic development, and special events. The researcher took on a friendly, least-adult role rather than authority figure (Kirk, 2006), and the children understood the researcher to be an adult volunteer who sometimes engaged with them but other times quietly observed them. This period of prolonged engagement and long-term observation, combined with rich, thick description and detail, adds to the credibility of the study findings.

Because the researcher is the primary data collection tool in ethnographic research, some bias could be introduced because of the subjectivity of the data collection/analysis process. However, engaging in the self-reflective process of “bracketing,” whereby the researcher recognizes their own *a priori* knowledge and assumptions and sets them aside in order to remain open to the participant's account (Gearing, 2004), was used to increase credibility. Specifically, the researcher took daily field notes that included personal memos to separate and reflect on her personal biases and *a priori* knowledge of the phenomenon of interest. As a physical activity and obesity researcher, the researcher understood that children aged 6–11 years engage in both physical activity and sedentary behaviors, and few meet recommended US guidelines of 60 min per day. The researcher recognized that children of immigrants face barriers related to low socioeconomic status that may have hindered physical activity engagement; however, engaging in an afterschool program may have promoted physical activity in this group. The personal memos provided the researcher with a space to begin to draw inferences from observations, discuss emerging themes, and link with other information (personal experiences or published literature), while keeping this information separated from the actual description of the event or situation being observed.

Finally, triangulation of sources (i.e., the use of multiple data sources and methods across time, space, and persons) (Graue & Walsh, 1998) was used to ensure a more complete understanding of the phenomenon. The reader is able to judge transferability of the findings based in the scrutiny of the data collection and analysis. Although the findings will likely not be applicable to Hispanic children of immigrants as a whole, the reader may decide the extent to which these findings can be transferred to other populations/groups with similar contexts and backgrounds.

### Measures

#### Child observations

Child observations including field notes during observation, were collected by the lead researcher. Daily jottings were collected each day in a small notebook, and field notes were transcribed each evening following the observation. Initial observations included recording field notes of the “everyday observable,” that is, taking everything in by observing the activities, settings, behaviors (Graue & Walsh, 1998), conversations, and interactions of the children as they pertained to physical activity. With time, the researcher focused on the preliminary patterns and themes that were emerging from the data record.

#### Semi-structured interviews

The researcher conducted semi-structured individual interviews with children to gather information about the child's physical activity attitudes and perceptions, context of, and barriers to their physical activity. Examples of questions included, “What do you like about physical activity?”; “What bad things would happen to you if you did not do physical activity?”; and “Is there anything that gets in the way of you being more physically active?” Prompts attempted to solicit information across levels of the Social Ecological model (e.g., parents, home, school, and neighborhood). Each child's interview took place in a private room at the afterschool location, was audio recorded, and lasted approximately 1 h.

#### Photo activity

PhotoVoice methods (Wang & Burris, 1997) were used to provide a deeper understanding of children's perceptions of and context for physical activity. Each child was given a disposable camera and instructed to take pictures over the course of 7 days (with the help of a parent if needed) of anything that they believed were related to their physical activity or play. Children were trained in the ethics of picture taking and instructed not to take pictures that would reveal the identities of individuals included in the photo. Children returned the cameras to the researcher, who developed the photos and scheduled a time to talk with the children individually about their photos. During this follow-up interview, the children reviewed the pictures with the lead researcher, described the picture, why it was special, and how it was related to their physical activity. Specifically, children were prompted to discuss more about who they were with and where the activity took place (i.e., physical and social context) to draw out information across levels of the Social Ecological model. Similar to the semi-structured interviews, each PhotoVoice interview took place in a private room at the afterschool location, was audio recorded, and lasted approximately 30–45 min.

#### Demographics

Demographic information was collected on participants, including child's sex, age, and child's/mother's/father's country of birth.

### Analysis

Field notes from child observations along with interview transcripts were compiled to form the complete data record. Data (i.e., field notes, jottings, interview transcripts, and photos) were triangulated and uploaded into NVivo9 (QSR International, Cambridge, MA, USA) to manage data analysis. Interview transcripts were analyzed and coded according to the constant comparison method (Glaser & Strauss, 1967) by the lead researcher. In this inductive process, each piece of data (e.g., sentence and paragraph) was compared and contrasted with other data to gain a greater understanding of the categories that existed within the phenomenon (i.e., Hispanic immigrant-origin children's perceptions of physical activity). The coding process began with “open coding,” where simple codes were given to discrete items/chunks of text. Specifically, the researcher read through the transcripts repeatedly line by line to identify “units” (aka codes) related to the research questions. Next, “axial coding” was performed by assigning categories based on patterns and relationships that were evident throughout the codes. This is a systematic thought process where individual “units” or codes at the specific level were clumped together into groups with shared characteristics. Finally, selective coding was performed whereby the central phenomenon or “core category” was identified and described, and all other categories were placed in relation to that phenomenon. Pictures that best represented the emergent themes and categories were selected and integrated into the final presentation of the data. Findings were presented to the lead researcher's dissertation committee members, which included three academics with extensive expertise in conducting qualitative research with underserved, marginalized, and international populations. Any discrepancies in the codes or categories were discussed and resolved as a team.

## Results

### Emergent themes and categories

In-depth, qualitative data analysis yielded four overarching themes regarding children's perspectives on physical activity (Table II). The following section details these key themes and core categories, and features selected quotes drawn from individual interviews with the children. Participant names have been replaced with aliases, and all photographs were used with participant permission.

**Table II:** Emergent themes and categories from qualitative data analysis.

Themes	Core categories	Categories
Theme 1. Activity across the spectrum: diverse types, locations, and people	Physical activities and contexts	Types of activities (physical activity and sedentary behavior) Physical context (where?) Social context (with whom?)
Theme 2. The good, the bad, and the ugly: what children really think about physical activity	Physical activity attitudes and perceptions	Perceived benefits of physical activity Negative attitudes toward physical activity
Theme 3. Play or physical activity? Either way, it's all about the fun	Physical activity as play	Physical activity is fun Creative play Physical sensations during play Resourcefulness despite a lack of resources
Theme 4. Helping or hindering forces: both at play in children's physical activity	Physical activity facilitators and barriers	Physical activity facilitators Physical activity barriers

#### Theme 1 - Activity across the spectrum: diverse types, locations, and people

Children reported and were observed engaging in both sedentary and physical activities. Playing video games was salient in boys’ discussions of popular activities, whereas girls generally reported playing computer games. All children reported watching television regularly, and many had a television in their bedroom and up to four televisions in their homes. Other popular sedentary behaviors included reading, drawing/painting, and playing with action figures or dolls. Some children characterized outdoor play as being more fun, because, as one child put it, “outside you can do a lot more stuff” (“Sebastian,” 8-year-old boy). Popular outdoor physical activities reported by children included hop scotch, climbing trees, playing tag, and using playground equipment. Riding skateboards (boys only), scooters, and bicycles were referenced throughout the interviews and photo discussions; however, negative associations emerged, including their bike was too small, broken, or had been stolen.

The most popular partners for activity included friends, parents, or other family members (e.g., siblings and cousins). Neighborhood friends were frequent companions for outdoor physical activities. Parents, especially fathers, were often cited by children as participating in physical activity with them (e.g., basketball at the park).

**Figure 1:**
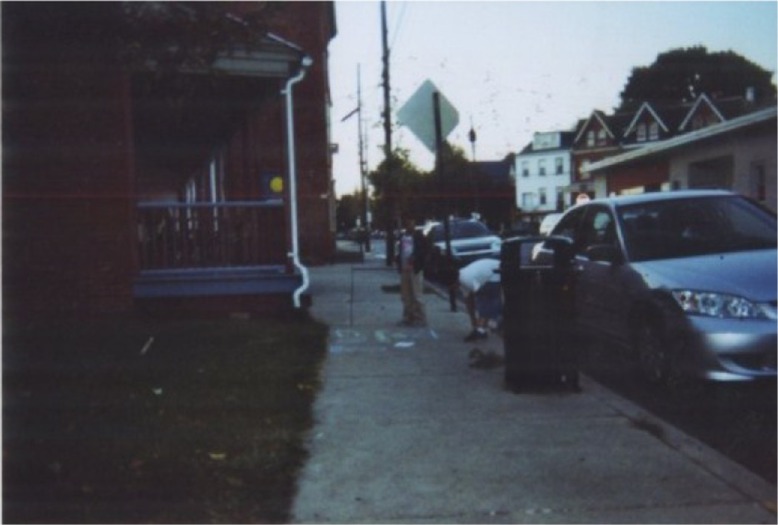
Sidewalk outside my home.

The most popular locations for physical activity included parks, school, the afterschool program, and inside/outside of their homes. Children would go to the park accompanied by their parents or older siblings; many reported that they were not allowed to go alone, because of the distance to the park or safety concerns of the parents. The gymnasium at the afterschool program was perceived as a place to run, get energy out, and have fun. Streets, sidewalks, and alleyways outside of the children's homes were places where they played sports (baseball, football, etc.), rode their bikes, scooters, and skateboards (Figure 1). However, these places often had heavy traffic and the children said they needed to be supervised by a parent.

#### Theme 2 - The good, the bad, and the ugly: what children really think about physical activity

**Figure 2:**
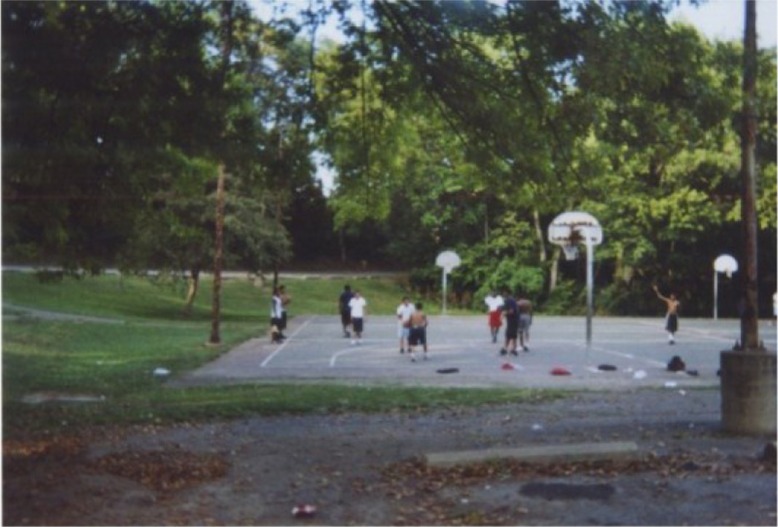
Playing basketball with friends.

Children perceived distinct physical, health, and social benefits of physical activity. Physical benefits included getting stronger, getting more muscles (boys only), and getting better at sports. One child, responding as to why he likes physical activity, said, “It helps you exercise and run more fast and shoot hoops more better” (“David,” 7-year-old boy). Health benefits included, “it helps your lungs” (“Jose,” 9-year-old boy) and it is “good for your heart” (“Pablo,” 10-year-old boy). The social benefits of physical activity were also evident in the interviews, with one child stating, “you have fun, and you make new friends” (“Adrian,” 9-year-old boy) (Figure 2).

Children's negative attitudes toward physical activity were related to physical discomfort, low athletic competence, and safety concerns. Physical discomforts were mentioned by almost every child as a major reason for disliking physical activity. One child shared, “Your legs get tired and your arms and stuff. Then you can't run anymore and you have to take breaks” (“Santiago,” 10-year-old boy). Sweating and smelling bad, mentioned by both boys and girls, was also a deterrent from physical activity. Some children mentioned that they disliked physical activity because they did not know how to play or were not good at specific activities (e.g., swimming, dancing, and soccer). Some children refrained from participating in certain activities for fear of getting hurt.

#### Theme 3 - Play or physical activity? Either way, it's all about the fun

Children perceived physical activity and play to be one in the same. For example, when asked what he likes about physical activity (i.e., any activity that makes him sweat or breathe hard and gets his heart pumping), one child responded, “I don't like sweating and breathing hard. I just like to ride my bike,” (“Luis,” 6-year-old boy) valuing play above all else. Furthermore, not only did children use the terms interchangeably, but “physical activity” and “play” were equally noted as fun.

**Figure 3:**
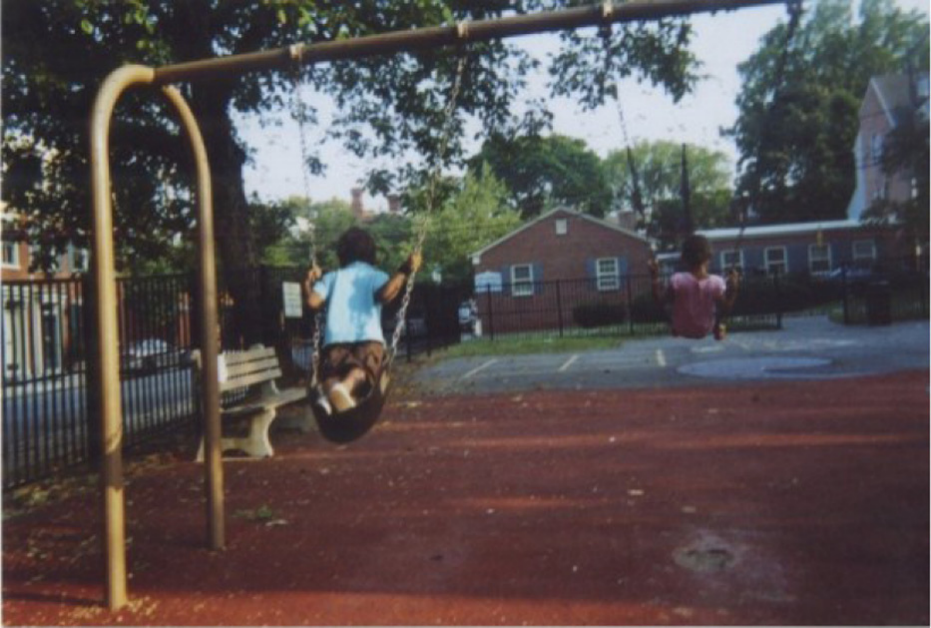
Swinging on the swings at the park.

Many children mentioned the physical sensations that were associated with doing fun activities. “[Swinging on the swings] can help you feel like you're flying and you're in the air…” (“Diego,” 7-year-old boy) (Figure 3). Hanging upside down and getting dizzy were other sensations the children enjoyed during their play. Furthermore, children engaged in creative play and would often make up games with their peers. The children were also resourceful despite a lack of resources (e.g., limited space or equipment). For example, one child shared, “My mom, sometimes she plays with me. Like we get a cup and a ball, and like, get a stick, and pretend like we're playing golf” (“Andrea,” 8-year-old girl).

#### Theme 4 - Helping or hindering forces: both at play in children's physical activity

The facilitators and barriers of children's physical activity were related to the home, school, and neighborhood environments.

### Physical activity facilitators

**Figure 4:**
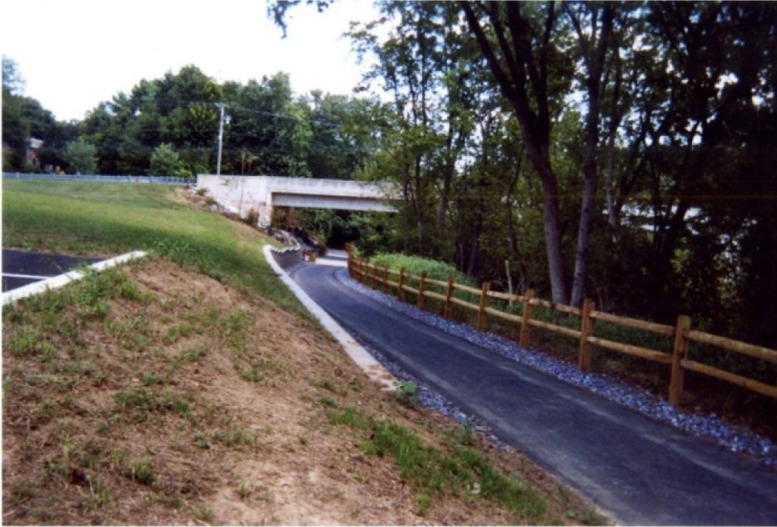
Walking/bike trail behind my house.

Parents were discussed as facilitators of the children's physical activity through modeling, encouraging children to play outside, signing them up for sports teams, and providing transportation. Sports team sign-ups were initiated by parents as well as requested by children. One child said, “I bring them like, the flyer, and they take me to that place, and then I get signed up, and then I get more physical [activity]” (“Samuel,” 11-year-old boy). The child's school context, including physical education class and recess, were also settings that promoted physical activity. Neighborhood resources, such as parks and bike paths, were cited as facilitators of children's physical activity. At the time of data collection, a greenway trail was built behind one child's home (Figure 4). When asked why his picture of the greenway was special he responded, “Because I – if the people didn't make the trail, then we'd still be riding our bike up [on the sidewalk]” (“Sebastian,” 8-year-old boy). The trail provided this child with a safe place to ride his bike, away from traffic, for an extended distance. The afterschool program, specifically the structured and unstructured physical activity that occurred in the gym, was also discussed as a facilitator. However, upon observation, the researcher learned that many girls were not allowed to play in the gym because they lacked appropriate clothing (e.g., sneakers).

### Physical activity barriers

**Figure 5:**
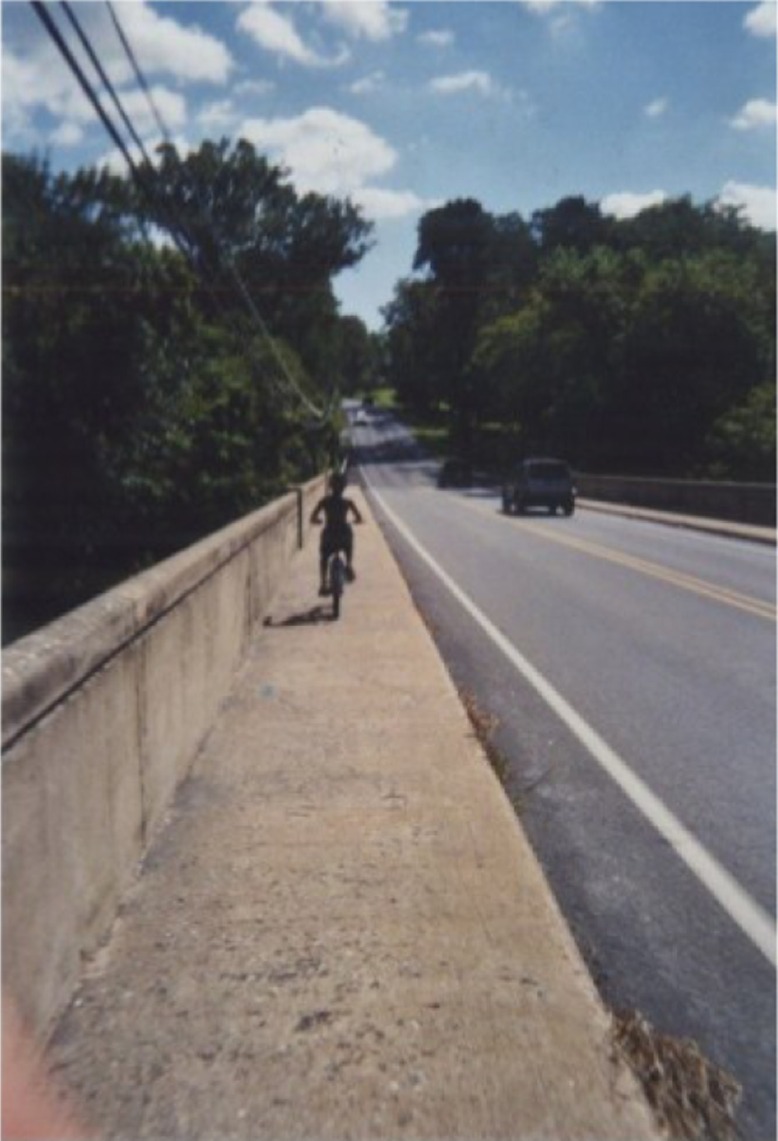
Riding bikes beside the road.

Some children mentioned that their parents’ work schedules (e.g., multiple jobs and odd hours) made their mom/dad tired and not able to participate in activities with them or take them to sports practice. For example, one child stated, “I have to go to practice after school and before school, and my grandpa doesn't know that. And my mom's always sleeping after school” (“Andrea,” 8-year-old girl). Also, a lack of space, including nowhere to run or play sports outside, was mentioned as a barrier. Although school physical education and recess were cited as facilitators of activity, children also shared that the school day in general was not conducive to being active. Furthermore, many complained that recess was too short, “We only get like, 10 minutes though” (“Santiago,” 10-year-old boy), and that physical activity was often withheld as punishment. Several neighborhood barriers were mentioned by children, including theft (e.g., bicycles stolen from home/park), lack of amenities at parks, and high cost of organized sports teams. Many children spoke about the barrier of heavy traffic and cars near their homes, which made it unsafe to play, “We can't play there because there's a lot of cars” (“Nicolas,” 11-year-old boy) (Figure 5).

## Discussion

The aim of this study was to describe 6- to 11-year-old Hispanic immigrant-origin children's perspectives regarding their physical activity perceptions, context, barriers, and facilitators. Children's physical activity patterns were partly determined by their own enjoyment, activity preferences, and availability. Corroborating previous studies, children reported engaging in both high levels of sedentary and physical activities (Biddle, Gorely, Marshall, Murdey, & Cameron, 2004; Centers for Disease Control and Prevention, 2014) Findings emphasized children's use of the terms “fun” and “play” over “physical activity” or “exercise”; further, opportunities for outdoor play, and unstructured, creative play were desirable in this population. This study identified facilitators and barriers of physical activity at all levels of Social Ecological Theory (i.e., personal, interpersonal, school, and community), supporting previous studies with other child populations (Ferreira et al., 2007; Gordon-Larsen, McMurray, & Popkin, 2000; Patnode et al., 2010; Sallis, Prochaska, & Taylor, 2000). The children perceived that their parents provided tangible support for physical activity through modeling, encouraging, signing them up for sports teams, and providing transportation (Horn & Horn, 2007; Kohl & Hobbs, 1998; Lindqvist, Kostenius, Gard, & Rutberg, 2015; Sallis et al., 2000; Trost et al., 2003; Welk, Wood, & Morss, 2003). The neighborhood barriers to physical activity mentioned by children (e.g., traffic, safety, theft, and lack of physical activity resources) were in line with commonly cited barriers in underserved communities (Amesty, 2003; Gordon-Larsen, Nelson, Page, & Popkin, 2006; Kumanyika & Grier, 2006). In this study, the afterschool program was likely a major facilitator of children's physical activity (Galvez et al., 2013; Trost, Rosenkranz, & Dzewaltowski, 2008), in that it afforded them a unique opportunity for safe, adult-supervised play within an underserved neighborhood.

### New contribution to the literature

The current study is unique in that it provides a glimpse into the experience of physical activity from the child's perspective, rather than relying on parent-proxy report, which is more common. This study complements and extends previous findings by including a sample of 6- to 11-year old, first- and second-generation Hispanic children and taking an in-depth, ethnographic approach by triangulating multiple sources of data over prolonged observation and engagement in the field. Given the low levels of physical activity in the first- and second-generation Hispanic children, and the other hardships they face regarding their experience as children of immigrants, this population's health is likely at risk. Based on a recent review of the literature, intervention studies that focus on health and well-being should include programs that facilitate physical activity behavior (Kristen, Ivarsson, Parker, & Ziegert, 2015). Information gleaned from this study can inform efforts of researchers and clinicians who seek to develop and implement programs to prevent obesity and promote physical activity in Hispanic immigrant-origin children. Therefore, this approach is an important and timely step to advocate for the health and well-being of this growing yet at-risk population.

### Future research

Although small with limited generalizability, this study may provide the groundwork for subsequent studies to further explore the underlying themes and patterns in the data. Based on the current findings, we recommend three key directions for future research: (1) studies that include a more diverse group of Hispanic immigrant-origin children to better understand the identified facilitators and barriers and any group differences (e.g., Hispanic subgroups, overweight/obese children, age, and gender); (2) studies that include a comparison group of children from other race/ethnicities to explore similarities and differences to Hispanic culture and values in physical activity perceptions; and (3) studies that include immigrant parents and their perspectives on physical activity to further extract meaning behind the findings of this study.

### Strengths and limitations

A major strength of this study is that it presents Hispanic children's perspectives of their physical activity through triangulation of multiple sources of data (field notes, transcripts, and photos) and in-depth analysis. This study was limited by the fact that it included a small sample of Hispanic children who were primarily second-generation boys from Puerto Rico and recruited from an afterschool program. Although the sample is small, we believe that we can glean information that can be transferred to Hispanic children living in urban environments, in general. Transferability (i.e., the ability to apply the results from Hispanic children in this study to other Hispanic children) is the goal of qualitative research. By providing rich description and detailed surrounding data collection and analysis, the reader is able to decide the extent to which these findings can be transferred to other populations/groups with similar contexts and backgrounds. Furthermore, the data collection and analysis were conducted by a sole researcher; however, in this study, trustworthiness was increased through previously mentioned techniques related to credibility and transferability (e.g., prolonged engagement, bracketing).

## Conclusions

In conclusion, this study provides important information about physical activity in Hispanic immigrant-origin children. Although the findings were specific to a small group of Hispanic children attending an afterschool program, it lays the groundwork for future studies to further explore some of the themes identified here. Moving forward, stakeholder engagement in future research and intervention development will be necessary and valuable. Findings from this study support the use of multi-component, multi-leveled interventions that focus on play in order to increase physical activity and ultimately promote health and well-being in this population.

## References

[CIT0001] Amesty S. C (2003). Barriers to physical activity in the Hispanic community. Journal of Public Health Policy.

[CIT0002] Biddle S. J, Gorely T, Marshall S. J, Murdey I, Cameron N (2004). Physical activity and sedentary behaviours in youth: Issues and controversies. The Journal of the Royal Society for the Promotion of Health.

[CIT0003] Bronfenbrenner U (1979). The ecology of human development: Experiments by nature and design.

[CIT0004] Bronfenbrenner U (1986). Ecology of the family as a context for human development: Research perspectives. Developmental Psychology.

[CIT0005] Bronfenbrenner U, Morris P. A, Damon W, Lerner R. M (1998). The ecology of developmental processes. Handbook of child psychology: Vol. 1. Theoretical models of human development.

[CIT0006] Butte N. F, Gregorich S. E, Tschann J. M, Penilla C, Pasch L. A, De Groat C. L (2014). Longitudinal effects of parental, child and neighborhood factors on moderate-vigorous physical activity and sedentary time in Latino children. International Journal of Behavioral Nutrition and Physical Activity.

[CIT0007] Butte N. F, Puyau M. R, Adolph A. L, Vohra F. A, Zakeri I (2007). Physical activity in nonoverweight and overweight Hispanic children and adolescents. Medicine and Science in Sports and Exercise.

[CIT0008] Capps R, Fix M, Ost J, Reardon-Anderson J, Passel J. S (2004). The health and well-being of young children of immigrants.

[CIT0009] Centers for Disease Control and Prevention (2014). Youth risk behavior surveillance–United States, 2013. Morbidity and Mortality Weekly Report.

[CIT0010] Creswell J. W (2007). Qualitative Inquiry and research design: Choosing among five approaches.

[CIT0011] Davison K. K, Birch L. L (2001). Childhood overweight: A contextual model and recommendations for future research. Obesity Reviews.

[CIT0012] Ferreira I, van der Horst K, Wendel-Vos W, Kremers S, van Lenthe F. J, Brug J (2007). Environmental correlates of physical activity in youth—A review and update. Obesity Reviews.

[CIT0013] Galvez M. P, McGovern K, Knuff C, Resnick S, Brenner B, Teitelbaum S. L (2013). Associations between neighborhood resources and physical activity in inner-city minority children. Academic Pediatrics.

[CIT0014] Gearing R. E (2004). Bracketing in research: A typology. Qualitative Health Research.

[CIT0015] Glaser B. G, Strauss A. L (1967). The discovery of grounded theory: Strategies for qualitative research.

[CIT0016] Gordon-Larsen P, McMurray R. G, Popkin B. M (2000). Determinants of adolescent physical activity and inactivity patterns. Pediatrics.

[CIT0017] Gordon-Larsen P, Nelson M. C, Page P, Popkin B. M (2006). Inequality in the built environment underlies key health disparities in physical activity and obesity. Pediatrics.

[CIT0018] Graue E, Walsh D. J (1998). Studying children in context: Theories, methods, and ethics.

[CIT0019] Hannay J, Dudley R, Milan S, Leibovitz P. K (2013). Combining photovoice and focus groups: Engaging Latina teens in community assessment, *American Journal of Preventive Medicine*,.

[CIT0020] Hernandez D. J, Hernandez D. J (1999). Children of immigrants: Health, adjustment, and public assistance. Children of immigrants: Health, adjustment, and public assistance.

[CIT0021] Horn T. S, Horn J. L, Tenebaum G, Eklund R. C (2007). Family influences on children's sport and physical activity participation, behavior, and psychosocial responses. Handbook of sport psychology.

[CIT0022] Kirk S (2006). Methodological and ethical issues in conducting qualitative research with children and young people: A literature review. International Journal of Nursing Studies.

[CIT0023] Kohl H. W, Hobbs K. E (1998). Development of physical activity behaviors among children and adolescents. Pediatrics.

[CIT0024] Kristen L, Ivarsson A, Parker J, Ziegert K (2015). Future challenges for intervention research in health and lifestyle research—A systematic meta-literature review. International Journal of Qualitative Studies on Health and Well-Being.

[CIT0025] Kumanyika S, Grier S (2006). Targeting interventions for ethnic minority and low-income populations. Future of Children.

[CIT0026] LeCompte M. D, Schensul J. J (1999). Designing & conducting ethnographic research Ethographer's Toolkit.

[CIT0027] Lincoln Y, Guba E (1985). Naturalistic inquiry.

[CIT0028] Lindqvist A. K, Kostenius C, Gard G, Rutberg S (2015). Parent participation plays an important part in promoting physical activity. International Journal of Qualitative Studies on Health and Well-Being.

[CIT0029] Liu J. H, Probst J. C, Harun N, Bennett K. J, Torres M. E (2009). Acculturation, physical activity, and obesity among Hispanic adolescents. Ethnicity and Health.

[CIT0030] Merianos A. L (2013). The skinny on physical activity interventions among Hispanic youth. Journal of Behavioral Health.

[CIT0031] Merriam S (1998). Qualitative research and case study applications in education.

[CIT0032] Mier N, Lee C, Smith M. L, Wang X, Irizarry D, Avila-Rodriguez E. H (2013). Mexican-American children's perspectives: Neighborhood characteristics and physical activity in Texas-Mexico border colonias. Journal of Environmental Health.

[CIT0033] Olvera N, Smith D. W, Lee C, Liu J, Lee J, Kim J. H (2011). Comparing high and low acculturated mothers and physical activity in Hispanic children. Journal of Physical Activity and Health.

[CIT0034] Patnode C. D, Lytle L. A, Erickson D. J, Sirard J. R, Barr-Anderson D, Story M (2010). The relative influence of demographic, individual, social, and environmental factors on physical activity among boys and girls. The International Journal of Behavioral Nutrition and Physical Activity.

[CIT0035] Patton M. Q (1990). Qualitative evaluation and research methods.

[CIT0036] Physical Activity Guidelines Advisory Committee (2008). Physical activity guidelines advisory committee report, 2008.

[CIT0037] Rawlins E, Baker G, Maynard M, Harding S (2013). Perceptions of healthy eating and physical activity in an ethnically diverse sample of young children and their parents: The DEAL prevention of obesity study. Journal of Human Nutrition and Dietetics.

[CIT0038] Sallis J. F, Prochaska J. J, Taylor W. C (2000). A review of correlates of physical activity of children and adolescents. Medicine and Science in Sports and Exercise.

[CIT0039] Schensul S. L, Schensul J. J, LeCompte M. D, Schensul J. J, LeCompte M. D (1999). Essential ethnographic methods: Observations, interviews, and questionnaires. Ethnographer's toolkit 2.

[CIT0040] Singh G. K, Kogan M. D, Yu S. M (2009). Disparities in obesity and overweight prevalence among US immigrant children and adolescents by generational status. Journal of Community Health.

[CIT0041] Singh G. K, Yu S. M, Siahpush M, Kogan M. D (2008). High levels of physical inactivity and sedentary behaviors among US immigrant children and adolescents. Archives of Pediatrics & Adolescent Medicine.

[CIT0042] Skelton J. A, Irby M. B, Guzman M. A, Beech B. M (2012). Children's perceptions of obesity and health: A focus group study with Hispanic boys, Infant. Child & Adolescent Nutrition.

[CIT0043] Snethen J. A, Hewitt J. B, Petering D. H (2007). Addressing childhood overweight: Strategies learned from one Latino community. Journal of Transcultural Nursing.

[CIT0044] Stokols D (1996). Translating social ecological theory into guidelines for community health promotion. American Journal of Health Promotion.

[CIT0046] Suarez-Orozco C, Yoshikawa H, Tseng V (2015). Intersecting inequalities: Research to reduce inequality for immigrant-origin children and youth.

[CIT0047] Taverno S. E, Rollins B. Y, Francis L. A (2010). Generation, language, body mass index, and activity patterns in Hispanic children. American Journal of Preventive Medicine.

[CIT0048] Taylor W. C, Yancey A. K, Leslie J, Murray N. G, Cummings S. S, Sharkey S. A (1999). Physical activity among African American and Latino middle school girls: Consistent beliefs, expectations, and experiences across two sites. Women and Health.

[CIT0049] Trost S. G, Rosenkranz R. R, Dzewaltowski D (2008). Physical activity levels among children attending after-school programs. Medicine and Science in Sports and Exercise.

[CIT0050] Trost S. G, Sallis J. F, Pate R. R, Freedson P. S, Taylor W. C, Dowda M (2003). Evaluating a model of parental influence on youth physical activity. American Journal of Preventive Medicine.

[CIT0051] Umstattd Meyer M. R, Sharkey J. R, Patterson M. S, Dean W. R (2013). Understanding contextual barriers, supports, and opportunities for physical activity among Mexican-origin children in Texas border colonias: A descriptive study. BMC Public Health.

[CIT0052] Wang C, Burris M. A (1997). Photovoice: Concept, methodology, and use for participatory needs assessment. Health Education & Behavior.

[CIT0053] Welk G. J, Wood K, Morss G (2003). Parental influences on physical activity in children: An exploration of potential mechanisms. Pediatric Exercise Science.

[CIT0054] World Health Organization (2010). Global recommendations on physical activity for health.

